#  A In Vitro and In Vivo Study of the Ability of NOD1 Ligands to Activate the Transcriptional Factor NF-kB 

**Published:** 2011

**Authors:** A.I. Tukhvatulin, D.Y. Logunov, I.I. Gitlin, M.M. Shmarov, P.V. Kudan, А.А. Adzhieva, A.F. Moroz, N.N. Kostyukova, L.G. Burdelya, B.S. Naroditsky, A.L. Gintsburg, A.V. Gudkov

**Affiliations:** Gamaleya Research Institute of Epidemiology and Microbiology, Russian Academy of Medical Sciences; Roswell Park Cancer Institute, Elm & Carlton Streets, Buffalo, New York, USA

**Keywords:** pattern-recognition receptors, Toll-like receptors, NOD1-receptor, transcriptional factor NF-kB, peptidoglycan of gram-negative bacteria

## Abstract

Pattern-recognition receptors (PRR) play a crucial role in the induction of the defense reactions of the immune system against pathogenic bacterial and viral infections. The activation of PRR by specific, highly conserved pathogen-associated molecular patterns (PAMPs) induces numerous immune reactions related both to innate and adaptive immunity. In addition to the well-studied Toll-like receptors, pathogens can be recognized by the receptors belonging to the other PRR families; including NOD-like receptors (NLR). Stimulation of members of NOD-like receptors (NOD1, 2) and Toll-like receptors results in the activation of the transcriptional factor NF-kB regulating gene expression in numerous molecules implicated in the development of proinflammatory reactions. As opposed to Toll-like receptors, the NF-kB-activating ability of NLRs has not been fully studied. In this work, we examine the ability of one member of the NLR family – NOD1 – to activate the main proinflammatory transcriptional factor NF-kB. We also compare the NF-kB-activating ability of NOD1 ligands of a different structure with TLR4,5 ligands*in vitro*and*in vivo*.

##  INTRODUCTION 

 Until recently, both the mechanisms of recognition of infectious agents by macro-organism cells, as well as the molecular mechanisms of innate and adaptive immunity as a response to the developing infection, had not been subjected to adequate study. 

An important step in the understanding of these mechanisms occurred twenty years ago, with the discovery of the first representatives of the pattern recognition receptors (PRR) of innate immunity: TOLL-like receptors (TLR). Today, seven PRR families are known: Toll-like receptors, NOD (nucleotide-oligomerization domain)-like receptors (NLR), RIG-like receptors (RLR), lectin-like receptors, etc. [2]. Representatives of these families can recognize a set of highly conservative fragments of exogenous pathogen-associated molecular pattern (PAMP) molecules, as well as some endogenous damage-associated molecular pattern (DAMP) molecules; each receptor having its specific set. The binding of intrinsic ligands with pattern-recognition receptors triggers intracellular signaling cascades, resulting in the activation of a number of transcriptional factors (AP-1, NF-kB, IRF 1, 3, 5, 7, etc.) which regulate the development of particular immune responses.

NF-kB is the primary pro-inflammatory factor that regulates the expression in a number of molecules which can participate in the development of reactions of both innate and adaptive immunity, such as the secretion of pro-inflammatory cytokines and chemokines, synthesis of antimicrobial peptides, induction of phagocytic activity of the microphages, dendritic cell maturation, etc. [3].

 Toll-like receptors and the representatives of the fairly recently discovered subfamily of NOD-like receptors, NOD1,2, belong to pattern-recognition receptors, the activation of which results in an NF-kB induction. 

Toll-like receptors are transmembrane proteins that are localized on the surface of the plasma membrane of cells and within intracellular compartments (endosomes). Such molecules of a different chemical nature, such as lipopolysaccharides (LPS), flagellin, bacterial lipopeptides, bacterial and viral DNA, etc., may serve as ligands of Toll-like receptors. Abundant data characterizing the ability of TLR ligands to activate NF-kB both *in vitro* and  *in vivo has * been collected [4]. The mediated NF-kB ability of TLR ligands to initiate different reactions of the immune system allows to use ligands as immediate protection agents against pathogens, molecular adjuvants, etc. [5–7].

Members of another PRR family – NOD receptors – are located directly in the cell cytoplasm and can recognize various molecules, which are fragments of peptidoglycan of gram-positive and gram-negative bacteria. It has been shown that NOD receptors participate in the recognition of bacteria capable of escaping the endosomal space and penetrating the cell cytoplasm and which can trigger specific immune responses [8]. In contrast to TLR ligands, the properties of NOD receptor ligands, including their ability to activate NF-kB, have been studied considerably less.

This work focused on a comparative in *vitro * and * in vivo* analysis of the NF-kB-activating ability of ligands of one representative of NOD receptors, NOD1, as well as TLR 4 and 5 ligands.

##  EXPERIMENTAL 

**Cell lines**

The NF-kB-activating ability of ligands of the NOD1 and Toll-like receptor 5 *in vitro* was studied using HEK293 cell lines (human embryonic kidney epithelial cells) and HCT116 (human colon carcinoma cells) expressing NOD1 in humans. The cells were cultivated in a DMEM medium with 10 vol % of fetal bovine serum (ref. number SV30160.03, Hyclone,United States); 1 mg/ml glutamine (ref. number F032, PanEco, Russia); 50 U/ml penicillin; and 50 µg/ml streptomycin (ref. number A065, PanEco, Russia) was added at 37°С, in an atmosphere containing 5% CO _2_ . The cells were seeded at a ratio of 1 : 6 on day 2, after the monolayer had been obtained.

**Ligands of NOD1 and Toll-like receptors**

The chemically synthesized NOD1 ligands, represented by samples of dipeptide *D-* Glu-mDAP(iE-DAP), tripeptide *L* -Ala *-D-* Glu- *L* -mDAP (Tri-DAP), the tripeptide covalently bound to a monosaccharide MurNAc *-L-* Ala *-D-* Glu *-L-* mDAP (M-Tri-DAP), and the molecule that contained the lauric acid fragment, Lauroyl *-* γ *-D-* Glu *-D-* mDAP (iE-DAP-C12), in addition to the minimal recognition sequence, were purchased from Invivogen, United States (ref. numbers: tlrl-dap, tlrl-tdap, tlrl-c12dap). LigandТLR4 – LPS ­– was purchased from Sigma-Aldrich, United States (ref. number L3024). Ligand TLR5 –flagellin was prepared according to the procedure described in [9].

**Extraction and Purification of Molecule**

GlcNAc-MurNAc- *L-* Ala *-D-* Glu *-L-* mDAP ** – the fragment of peptridoglycane of** *Neisseria meningitidis* **serogroup В – NOD1 receptor ligand**

Primary cultivation of *N. meningitidis* (wild-type strain 591 belonging to serogroup B was isolated from the cerebrospinal fluid of a meningitis patient in Moscow in 1985. Phenotype: B : NT : P1.1,2) was carried out at a temperature of 37°C in the presence of 5% CO _2 _ in the Thayer Martin medium (ref. number M413, Himedia, India). In order to add selective properties to the medium, a set of antibiotics V.C.N.T. Supplement (ref. number FD024, Himedia, India) was introduced. The preparative growth of the bacterium was carried out in a Brain Heart Infusion broth (ref. number 211059, Difco, United States) with 10 vol % of equine serum at 37°C added upon rocking (160 oscillations/min). The ligand of a NOD1 receptor was extracted and chromatographically purified according to the earlier published procedure [10], which include the preliminary thermal inactivation of bacteria at 65°C for 1 h and phenol-chloroform extraction upon heating and mixing of the phenol–water mixture at 70°C for 30 min. The aqueous phase containing peptidoglycan fragments was subjected to tangential-flow ultra filtration (Millipore, United Stated) through a filter with a pore diameter of 30 kDa. The filtrate was purified via three sequential methods of reversed-phase high-performance liquid chromatography (HPLC) on a Bio-alliance 2796 chromatograph (Waters, United States). The first two stages of the purification procedure were carried out using a Symmetry С18 Column with a particle diameter of 5 µm, 4.6 × 75 mm (Waters, United States). During the final stage of purification, a Synergi Hygro-RP 80 Å column (particle diameter of 4 µm, 250 × 3 mm (Phenomenex, United States)) was used.

 Chromatographic separation of the compounds was performed on a spectrophotometric detector 2487 (Waters, United States, λ 205, 260, and 280 nm). A Q-STAR Elite mass spectrometer with Turbo Spray and Nano Spray ion spray ionization sources (Applied Biosystems/MDS SCIEX, United States) was used as a detector at the final (third) stage of purification. The resulting sample containing a mass spectrometrically pure substance underwent mass spectrometric analysis in order to determine the primary structure of the molecule under study. At the first stage of analysis, the accurate molecular mass of the substance, equal to 850.353 Da, was determined. The molecules of the substance subjected to study were then fragmented in an ion spray ionization source for further determination of the sequence of amino acids comprising the molecule, using Protein Pilot software (Applied Biosystems, United States). The non-protein part of the molecule was identified by pseudo-MS3 (studying the products of the secondary fragmentation of ions obtained by pre-decomposition of an analyzed molecule in the ionization source). The final brutto formula of the analyzed molecule was determined by a study of the decomposition paths of the decomposition of its ion. 

The comparative analysis of the fragmentation spectra of different control substances (including N-acetylglucosamine) and the analyzed compound under identical conditions made it possible to identify the primary structure of the molecule: GlcNAc-MurNAc- *L-* Ala *-D-* Glu *-L-* mDAP.

 The same experiment was performed on the samples of the bacterial culture medium (negative control) in order to verify the results obtained. Furthermore, the biological activity of the sample containing the substance under study, i.e., its ability to activate NF-kB in cells via the Toll-independent mechanism, was measured following each chromatographic stage of purification. 

**Measurement of β-galactosidase activity**

Twenty-four hours after the study samples were added to the cells, the culture medium was removed and the cell lysis buffer with β-galactosidase substrate (1 mM MgCl _2_ ; 0.25 M Tris-HCl, pH 7.4; 0.02% NP40; 2 g/l *o* -nitrophenyl-β- *D* -galactopyranoside (ref. number 102473, MP Biomedicals, United States)) was added. The level of activity of β-galactosidase was determined spectrophotometrically (414 nm) on the basis of substrate ( *o* -nitrophenyl-β- *D* -galactopyranoside) conversion into the colored product *o* -nitrophenol.

**Determination of bioluminescence intensity in HCT116 and HEK293 line cells containing the luciferase reporter gene under expression control of the NF-kB-dependent element**

At the preliminary stage, HCT116 and HEK293 line cells were infected with a lentiviral vector containing the luciferase reporter gene under expression control of the NF-kB-dependent promoter. A day before the bioluminescence was to be determined, the cells were seeded into a 96-well plate with a confluence of 2 × 10 ^4 ^ per well. The following day, the samples of PRR ligands were added to the cells, and after 8 more h, the lysis buffer containing luciferin Bright-Glo™ Luciferase Assay System (ref. number E2620, Promega, United States) was also added. The buffer volume (100 µl) was equal to the volume of the culture medium. The level of activity of luciferase was determined on the basis of the fluorescence intensity by means of a Wallac 1420 plate reader (Perkin Elmer, United States).

**Determination of bioluminescence intensity in the samples of organ homogenates of BalB/C transgenic mice**

 PRR ligands (LPS or iE-DAP-C12) were intramuscularly introduced into BalB/C transgenic mice containing the luciferase gene, under control of the NF-kB-dependent promoter, after which the organs were collected at intervals of 1, 3, and 5 h. The organs were homogenized in a Reporter Lysis 5x Buffer (ref. number E3971, Promega, United States) with inhibitors of cell proteases added. The level of activity of the transcriptional factor NF-kB in the samples of mouse organ homogenates was determined by the bioluminescence intensity in the samples, normalized with respect to protein content (10 mg) using a Wallac 1420 plate reader (Perkin Elmer, United States). 

**Determination of expression of NOD1 receptor by RT-PCR**

The total RNA from HCT116 and HEK293 cells was isolated using a Trizol reagent (ref. number 15596026, Invitrogen, United States), in accordance with the manufacturer’s procedure. A Superscript III kit (ref. number18080200, Invivogen United States) was used for cDNA synthesis. The amount of cDNA encoding the NOD1 receptor was normalized with respect to the amount of GAPDH cDNA. Thirty-five PCR cycles were performed with the use of primers specific to the nucleotide sequence of the cDNA sequence that encodes human NOD1 – NOD1-forw: ctt-ctg-gtc-act-cac-atc-cgc-a, NOD1-rev: tgg-gca-tag-cac-agc-acg-aac. The primer annealing temperature was measured at 62 ^о^ С.

**Real-time measurement of bioluminescence intensity in the organism of BalB/С transgenic mice**

The activity of the NF-kB factor in the organism of BalB/С transgenic mice containing the luciferase reporter gene in their genome, under expression control of the NF-kB-dependent promoter, was measured in real time on the basis of the bioluminescence intensity at intervals of 1, 3, and 5 h after PRR ligands (LPS or iE-DAP-C12) were introduced. Five minutes prior to obtaining the images, 1.5 mg of *D* -luciferin (Caliper Life Sciences) was injected into the mice. The bioluminescence intensity was determined on an IVIS Imaging System 100 instrument (Xenogen Corp., United States).

**Laboratory animals**

**Fig. 1:**
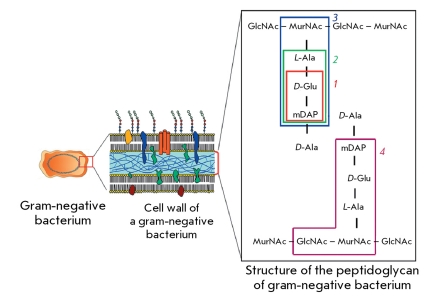
NOD1 ligands in the peptidoglycan structure of Gram-negative bacteria. *1 – D-Glu-mDAP (* iE-DAP *)* , *2* – *L-Ala-D-Glu-L-mDAP* (Tri-DAP), *3 – MurNAc-L-Ala-D-Glu-L-mDAP* (M-Tri-DAP), and *4* – *GlcNAc-MurNAc-L-Ala-D-Glu-L-mDAP * (GM-Tri-DAP). *GlcNAc * –N-acetylglucosamine, *MurNAc* – N-Acetylmuramic acid, *L-Ala * –L-alanin *, D-Glu * – *D-* Glutamic acid, *L-mDAP * – L-meso-Diaminopimelic acid.

 Female transgenic mice of the BALB/c-Tg(NFkB-RE-luc[Oslo])-Xen line (Caliper Life Sciences, United States) with a weight range of 18–20 g containing the reporter gene of firefly luciferase in their genome (from pGL3-Basic Vector, Promega, United States), under expression control of the NF-kB-dependent promoter consisting of three NF-kB-binding regions in the promoter of the Igκ light chain gene, were used for the purposes of this experiment. 

 The mice were given free access to water and food. LPS (5 µg/mouse) and iE-DAP-C12 (200 µg/mouse) solutions were injected intramuscularly into the mice. 

##  RESULTS AND DISCUSSION 

**Comparison of the NF-kB-activating ability of NOD1 receptor ligands under**

*in vitro* **conditions**

Dipeptide *D-* Glu-mDAP is known as the key sequence necessary and sufficient to inactivate the NOD1 receptor upon the decomposition of gram-negative bacteria and penetration of peptidoglycan fragments inside eukaryotic cells [10]. The participation of other amino acids and monosaccharides, the components of the structural molecules of peptidoglycan of gram-negative bacteria, in the recognition by the NOD1 receptor has yet to be described thoroughly ( *Fig. 1* ).

 It thus follows that the study of the dependence of NOD1-mediated NF-kB-activating ability of various peptidoglycan fragments on their structure seems of importance. 

**Fig. 2:**
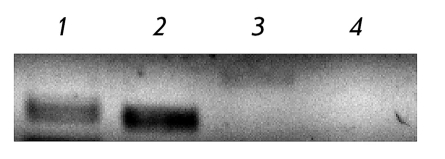
Expression levels of NOD1 receptor detected by RT-PCR with specific primers in *1 * – human colon carcinoma cells HCT116, *2 * – human embryonic kidney cells HEK293, *3 –* non-small cell lung carcinoma cells H1299, and *4* – human lung carcinoma cells A549.

In this context, we carried out a screening experiment at a preliminary stage to select the cell model in which the expression of the NOD1 receptor by different cell lines was determined. According to the RT-PCR data, expression of the NOD1 receptor was detected in both the HEK293 cell line (human embryonic kidney epithelial cells) and the HCT116 cell line (human colon carcinoma cells). TLR5 was also expressed in this cell line [11], whereas in Н1299 and A549 cell lines the expression of the NOD1 receptor could not be detected under the selected RT-PCR conditions ( *Fig. 2* ).

 Considering that HEK293 line cells do not express TLR (their presence may result in the additional activation of NF-kB when using NOD1 receptor ligands containing the impurities of TLR ligand molecules), this cell line served as a model for studying the ability of NOD1 receptor ligands possessing different structures to activate NF-kB. 

 Using the lentiviral vector, the gene of the β-galactosidase reporter protein under the transcriptional control of the NF-kB-dependent promoter and the marker of resistance to blasticidin S were introduced into the genome of HEK293 cells. The principle of use of the resulting cell line is based on the ability of the NOD1 receptor after its interaction with specific ligands to activate the kinase cascade (RIP2, IKKα,β), which results in the activation of the NF-kB factor, followed by its translocation into the nucleus, where this factor binds with the intrinsic regulatory element which controls the galactosidase gene. Hence, the activity of NF-kB in cells can be quantitatively determined on the basis of the staining reaction for galactosidase by registering the interaction between the NOD1 receptor and the intrinsic ligands. 

**Fig. 3:**
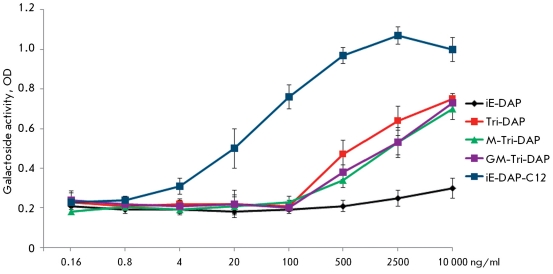
Activity of NF-kB in HEK293, expressing NOD1. NF-kB-dependent β-galactozidase activity was detected by spectrophotometric measurement (wavelength 414nm) of optical density of colored solution containing the converted specific substrate ortho-Nitrophenyl-β-galactoside (ONPG). The concentration of NOD1 receptor ligands is plotted along the X axis.

In order to measure the NF-kB activity, NOD1 receptor ligands with a different structure were added to HEK293 line cells: dipeptide *D-* Glu-mDAP (iE-DAP), tripeptide *L-* Ala *-D-* Glu *-L-* mDAP (Tri-DAP), and tripeptide MurNAc *-L-* Ala *-D-* Glu *-L-* mDAP (M-Tri-DAP) covalently bound with a monosaccharide. Furthermore, in our work we used the covalently bound with disaccharide tripeptide GlcNAc-MurNAc *-L-* Ala *-D-* Glu *-L-* mDAP(GM-Tri-DAP), prepared from *N. meningitidis * serogroup B and purified to mass spectrometrical purity using the earlier described method [10]. We additionally used a chemically synthesized molecule which contained the lauric acid fragment Lauroyl *-* γ *-D-* Glu *-D-* mDAP (iE-DAP-C12) capable of changing the physicochemical properties of this molecule, in addition to the minimal recognition sequence.

As can be seen in *Fig. 3* , a reliable increase in the NF-kB-dependent activation of the β-galactosidase gene expression was attained only at maximum concentrations (1–10 µg/ml) of the *D-* Glu-mDAP dipeptide added, whereas derivatives of this dipeptide with a higher molecular weight (Tri-DAP, M-Tri-DAP, and GM-Tri-DAP) than those that can be found as components of peptidoglycan of gram-negative bacteria induced a reliable increase in NF-kB-dependent β-galactosidase gene expression at lower concentrations (from 0.5 µg/ml). The maximum NOD1-mediated ability of NF-kD activation was shown by the chemically synthesized molecule iE-DAP-C12 *. * The minimum concentration of this molecule, which was capable of inducing the NF-kB-dependent activation of β-galactosidase gene expression, is equal to 20 ng/ml. Such a significant increase in the NF-KB-activating ability (by a factor of approximately 1,000 as compared with *D-* Glu-mDAP) can be explained by the presence of the hydrophobic component of lauric acid, which is likely to facilitate the penetration of iE-DAP-C12 molecule through the plasma membrane and binding to the NOD1 receptor located in the cell cytoplasm.

This experiment thus demonstrated that an increase in the amino acid sequence in the structure of the NOD1 receptor ligand results in a reliable increase in the NF-kB-activating ability of the molecule as compared with the NF-kB-activating minimal sequence *D* -Glu-mDAP, whereas the presence of monosaccharide residues within the ligand molecule does not further increase the activity.

 This phenomenon can be explained as follows. For an optimally efficient recognition by the NOD1 receptor (in order to penetrate into the “pocket” of its ligand-recognizing domain), a ligand molecule has to contain at least three covalently bound amino acids, whereas the additionally introduced saccharide molecules do not participate in the direct formation of the ligand-receptor complex. Moreover, the NF-kB-activating ability of the NOD1 receptor ligand can be significantly increased (by a factor of approximately 1,000) by the enhanced hydrophobicity of the molecule, as a result of binding with a fatty acid residue. In all likelihood, such a modification allows to speed up the process, ensuring the necessary ligand concentration near the receptor in the cytoplasm, because its penetration through the cell plasma membrane is made easier. 

**Fig. 4:**
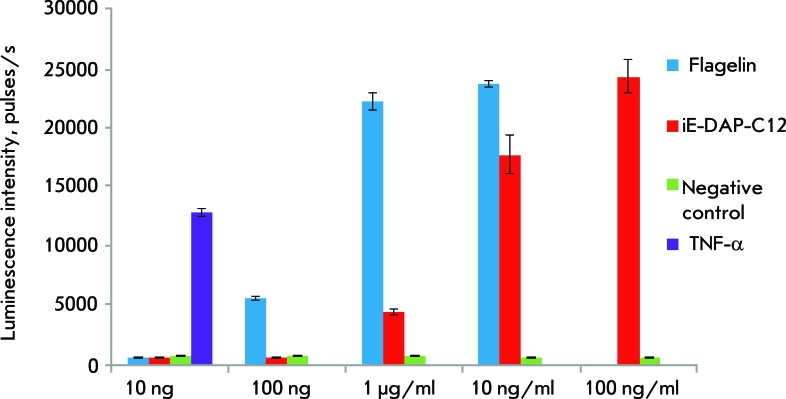
NF-kB activity in HCT116 expressing NOD1 and TLR5. ■ – cells treated with TLR5 ligand – flagellin; ■– cells treated with NOD1 ligand – iE-DAP-C12; ■ – control intact cells; ■ – cells treated with TNF-α (concentration 10 ng/ml). NF-kB-dependent luciferase activity was detected by spectrophotometric measurement of bioluminescence after addition of specific substrate - luciferin. Y-direction – luminescence intensity units (CPS).

 The iE-DAP-C12 ligand of the NOD1 receptor, which exhibited the highest activity, was selected for further studies. 

**Comparison of the NF-kB-activating ability of NOD1 and TLR5 ligands in human colon carcinoma cells HCT116**

In order to compare the NF-kB-activating ability of NOD1 and TLR5 ligands under *in vitro* conditions, the HCT116 cell line was selected as a model. This cell line expresses both the NOD1 receptor and TLR5, in which the bacterial flagellar protein flagellin serves as a ligand.

 The NF-kB activity was measured on the basis of the expression of the luciferase gene, which was introduced into the cell genome under the transcriptional control of the NF-kB-dependent element. 

 TNF-α was used as positive control of NF-kB activation. This cytokine stimulates NF-kB activation and induces the expression of proinflammatory factors after its interaction with the intrinsic receptor. 

The experimental results demonstrate ( *Fig. 4* ) that the most active NOD1 receptor–ligandiE-DAP-C12-–has an effect on NF-kB that can be compared with that of flagellin, but in concentrations that are higher by a factor of 10–100 than those of flagellin. In light of the results of this experiment, a conclusion can be drawn that NOD1 receptor ligands activate NF-kB to a lesser degree than TLR5 ligand; at least for the delivery path that was used. This phenomenon can be explained by a number of reasons: the fact that the NOD1 receptor ligands need to overcome additional barriers, such as the plasma membrane and the cytoplasmic space; a different affinity of TLR and NOD receptors to the intrinsic ligands; and the differences in the lower-lying signaling cascades, resulting in the activation of the transcriptional factor NF-kB.

 The ability of NOD1 receptor ligands to induce specific immune responses upon a weaker NF-kB activation in eukaryotic cells as compared with TLR could be an indication that the level of NF-kB activity may determine the development of particular specific immune responses and control their intensity. 

The established ratio between the concentrations of NOD1 and TLR ligands which activate NF-kB was taken into consideration to the same extent when carrying out the subsequent studies in *in vivo * conditions.

**Studying the NF-kB-activating ability of the NOD1 receptor ligand under**

*in vivo* **conditions**

It has been shown that the level of NF-kB activation may differ in different organs of laboratory animals into which TLR ligands has been injected [12, 13]. However, to date there has been no data that clearly shows the ability of the ligands of other PRR–NOD receptors to induce NF-kB activation under * in vivo * conditions. We investigated this question by determining the major activation parameters (the kinetics and intensity of activation in different organs) upon introduction of the NOD1 receptor ligand iE-DAP-C12, which exhibited the highest activity in *in vitro * experiments. Furthermore, we compared the NF-kB-activating abilities of NOD ligands and those of TLR4, the thoroughly studied Toll-like receptor, under * in vivo* conditions upon intramuscular injection.

**Fig. 5:**
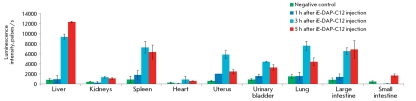
NF-kB activity in mouse organ homogenates 1,3,5 h after iE-DAP-C12 administration. Samples were normalized with respect to concentration of total protein (10mg/ml). ■ –NF-kB activity in organs of control intact mice 3 h after PBS injection. ■ – NF-kB activity in organs of mice 1 h after iE-DAP-C12 injection (200 µg/animal). ■ – NF-kB activity in organs of mice 3 h after iE-DAP-C12 injection (200 µg/animal). ■ – NF-kB activity in organs of mice 5 h after iE-DAP-C12 injection (200 µg/animal). NF-kB-dependent luciferase activity was detected by spectrophotometric measurement of bioluminescence after addition of specific substrate - luciferin. Y-direction – luminescence intensity units (CPS).

To this end, we used BalB/C transgenic mice with the luciferase reporter gene incorporated into their genome, under the control of a NF-kB-depending promoter. Such a system allows to measure the degree of NF-kB activation in different mouse organs, on the basis of luminescent glowing upon the introduction of PRR ligands [14].

In order to compare the parameters of the NF-kB activation that is observed upon the introduction of iE-DAP-C12, we selected LPS, the ligand of the Toll-like receptor that has been best studied under *in vivo * conditions [13, 15, 16].

The amounts of NOD1 and TLR4 ligands were selected based on the data on the NF-kB-activating ability of these ligands under *in* *vitro* conditions (200 µg/mouse and 5 µg/mouse, respectively).

It was demonstrated ( *Fig. 5* ) that upon the intramuscular introduction of iE-DAP-C12, the highest relative and absolute inductions of NF-kB activation (over 12 times) take place in the liver. Moreover, in the liver and the small intestine the highest degree of NF-kB activation is attained 5 h after the introduction of a ligand, whereas a decrease in the level of NF-kB activation was observed at that time in other organs. In three out of nine organs of laboratory animals (kidneys, heart, and small intestine), the introduction of the NOD1 ligand resulted in a minimum increase in the NF-kB activation, as compared with the level of NF-kB activation in the control group.

 At the final stage, we compared the levels of NF-kB activation in different organs of mice which received intramuscularly iE-DAP-C12 and LPS 3 h after the introduction of the ligands (maximum NF-kB activation point). 

**Fig. 6:**
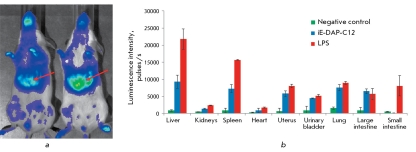
a - Real-time *in vivo* imaging of NF-kB activity in mouse organism after administration of iE-DAP-C12 (left) and LPS of * E. coli* (right). Red arrows point to the maximal bioluminescence intensity detected in liver after PRR ligands exposure. b -NF-kB activity in mouse organ homogenates 3 h after iE-DAP-C12 and LPS administration. Samples were normalized with respect to the concentration of total protein (10 mg/ml). ■ – NF-kB activity in organs of control intact mice 3 h after PBS injection. ■ – NF-kB activity in organs of mice 3 h after iE-DAP-C12 injection (200 µg/animal). ■ – NF-kB activity in organs of mice 3 h after LPS injection (5 µg /animal). NF-kB-dependent luciferase activity was detected by spectrophotometric measurement of bioluminescence after addition of the specific substrate - luciferin. Y-axis – luminescence intensity units (CPS).

As can be seen in *Figs. 6a and 6b* , the introduction of LPS results in a stronger NF-kB activation in a number of organs (liver, spleen, uterus, and small intestine), when compared with iE-DAP-C12. In the large intestine, lungs, and urinary bladder, the introduction of NOD1 and TLR4 ligands induced a comparable level of NF-kB activation. In other organs such as kidneys and the heart, no reliable increase in the level of NF-kB activation was observed as a response to the introduction of both iE-DAP-C12 and LPS.

Thus, according to the results of the study of the ability of NOD1 ligands to activate NF-kB under *in vivo * conditions, a conclusion can be drawn that the intensity and kinetics of NF-kB activation in response to the introduction of these ligands differ in different mouse organs. Furthermore, differences in the intensity of NF-kB activation in mouse organs after the introduction of NOD1 and TLR4 ligands in the selected concentrations were detected.

 After summarizing the data obtained, a conclusion can be drawn that the NF-kB factor activation induced by the interaction between the NOD1 receptor with intrinsic ligands differs from that upon stimulation of TLR representatives (kinetics and intensity of activation) in the same organ, which could be essential for the development of the subsequent immune responses. Moreover, the demonstrated tissue- and organ-specificity of NF-kB upon the introduction of Toll- or NOD receptor ligands into the organism of an animal may lead to the development of different local immune responses in a particular organ or tissue (e.g., differences in the spectra of secreted cytokines may be observed). The development of some immune responses can be accounted for by the distinctions in differentiation and cell types representing a tissue in which the induction of NF-kB activation took place. 

The ability of NOD1 receptor ligands to activate the NF-kB factor under *in vivo* conditions can be used for creating new molecular adjuvants which are characterized by a lower reactogenecity in comparison with some TLR ligands (e.g., LPS). Such adjuvants could be used together with vaccine antigens for protection against different pathogens, including intracellular ones. In order to bolster this hypothesis further, a detailed analysis of the individual immune responses that are initiated by the activation of NOD receptors is required.
